# From Multisensory Assessment to Functional Interpretation of Social Behavioral Phenotype in Transgenic Mouse Models for Autism Spectrum Disorders

**DOI:** 10.3389/fpsyt.2020.592408

**Published:** 2020-11-19

**Authors:** Hiroyuki Arakawa

**Affiliations:** Department of Psychology, Tokiwa University, Mito, Japan

**Keywords:** autism, mouse models, social deficits, phenotyping, behavior validity, sociability

## Abstract

Autism spectrum disorder (ASD) is a common heterogeneous disorder, defined solely by the core behavioral characteristics, including impaired social interaction and restricted and repeated behavior. Although an increasing number of studies have been performed extensively, the neurobiological mechanisms underlying the core symptoms of ASD remain largely unknown. Transgenic mouse models provide a useful tool for evaluating genetic and neuronal mechanisms underlying ASD pathology, which are prerequisites for validating behavioral phenotypes that mimic the core symptoms of human ASD. The purpose of this review is to propose a better strategy for analyzing and interpreting social investigatory behaviors in transgenic mouse models of ASD. Mice are nocturnal, and employ multimodal processing mechanisms for social communicative behaviors, including those that involve olfactory and tactile senses. Most behavioral paradigms that have been developed for measuring a particular ASD-like behavior in mouse models, such as social recognition, preference, and discrimination tests, are based on the evaluation of distance-based investigatory behavior in response to social stimuli. This investigatory behavior in mice is regulated by multimodal processing involving with two different motives: first, an olfactory-based novelty assessment, and second, tactile-based social contact, in a temporally sequential manner. Accurate interpretation of investigatory behavior exhibited by test mice can be achieved by functional analysis of these multimodal, sequential behaviors, which will lead to a better understanding of the specific features of social deficits associated with ASD in transgenic mouse models, at high temporal and spatial resolutions.

## Introduction

Autism spectrum disorder (ASD) is a heterogeneous neurodevelopmental disorder, the neuronal regulatory mechanisms underlying which requires further understanding (1, 2). Numerous studies on the clinical and neuropathological aspects of ASD have been pursued and several research strategies have been employed to investigate and elucidate the neurobiological mechanisms underlying ASD, including the utilization of animal models. Over the last two decades, studies using animal models, particularly transgenic mouse models, have contributed to promoting our understanding of genetic and neuronal processes in ASD. However, a large body of specific characteristics underlying ASD pathology and neurobiology remain to be uncovered (3, 4). This review provides an overview of recent trends and strategies employed in ASD studies, particularly with respect to the bottlenecks and caveats involved in research using mouse models, and proposes a key strategy for employing transgenic mouse models with modification of a single gene or chromosomal region for ASD research (5, 6).

### Clinical and Neurobiological Characteristics of ASD

The standard diagnostic manual, DSM-5, defines ASD as a neurobehavioral disorder manifested by persistent deficits in social and communicative interactions that involve understanding and maintaining social relationships, as well as abnormal and fixed interests and repetitive behavior (DSM-V, 2013). Although the exact etiology is largely unknown, these core symptoms can be observed before the age of 3 years and may persist throughout the entire lifetime (7, 8). Family and twin studies have provided accumulative evidence for the involvement of genetic factors in ASD (9–11), and genome-wide scans in ASD patients have indicated that there are several predisposing genes in susceptibility loci (12, 13). In addition to complex genetic susceptibility and interactions between multiple candidate genes for ASD, epigenetic changes, such as those resulting from exposure to environmental factors, are also responsible for ASD pathology, including the etiology and mechanisms underlying ASD (14, 15). Several clinical studies have illustrated that the risk of developing ASD is ~40% due to genetic variability, and the remaining 60% is caused by environmental (epigenetic) factors exposed during prenatal to postnatal periods (16–19).

The prevalence of ASD has markedly increased since the 1990s; it was 10–20 per 10,000 children worldwide (20, 21), and currently ASD affects 1 in 68 children in the United States (22). To date, there are no efficient therapeutic interventions that target the core symptoms of ASD (23, 24), although behavioral interventions produce significant results in some cases (25, 26). The neurobiological mechanisms underlying the pathology of ASD that mediates a primary symptom are poorly understood (27). There is an unquestionable need to elucidate the brain mechanisms responsible for regulating social behaviors, including identifying the specific neuronal circuitry and the transmission chemicals involved in these processes in ASD (28). This is mostly because of the difficulty in studying fundamental neurophysiological processes in the human brain. Therefore, findings using animal models expressing similar behavioral characteristic as humans are crucial for gaining a better understanding of the brain mechanisms involved in ASD, which would promote further research leading to an optimized therapeutic strategy, and thus, cure of the disease (28, 29).

### From Genetic to Behavioral Studies in Mouse Models of ASD

Animal studies allow coherent investigations of the cells, neural circuits, and pathophysiological processes relevant to ASD (30). Therefore, recent ASD research has changed focus from behavioral observations of symptoms to translating findings from animal models through the use of pharmacological and genomic manipulations, in order to reverse the symptoms relevant to ASD (31). There are broadly two types of animal models for ASD: etiology-driven models, in which environmental factors that cause ASD-like pathological processes are examined by exposure to certain chemicals or infections during pre- to post-natal, early developmental periods (32), and genomic-driven models, in which gene factors relevant to ASD pathology are investigated using transgenic manipulations in animals (6, 29, 33). The etiology-driven models are based on epidemiological evidence that early-life chemical exposures may be etiologically involved in certain symptoms of ASD (34, 35). Animal models could provide an answer to the question of whether these chemical exposures would be able to induce ASD-like behaviors and neural modifications in animals that mimic human symptoms (29, 36). The genomic-driven models can elucidate ASD-relevant pathology and mechanisms that are a result of mutations in single or multiple genes (6, 28).

The genetic basis of ASD has been consistently demonstrated since early studies (16, 37). Most of the known genetic alterations contributing to increased ASD risk affect the expression or function of proteins with established roles in the formation, function, and maintenance of synapses/neurons or in chromatin remodeling (6). Accordingly, distinct human genetic diseases relevant to ASD are caused by a specific single gene mutation (1, 33). The genetic manipulation of target genes in animal models of ASD would be expected to exhibit behavioral phenotypes reminiscent of human ASD, such as impaired social interaction and communication, and restricted and repetitive behaviors (6). The complex neural organization underlying social interaction has been daringly investigated in rodent (particularly mouse) models, which have allowed researchers to delve into exhaustive mechanistic depth in the neural circuity of genetically manipulated species that are otherwise highly social (29). Several research groups have developed mouse models of ASD, driven by a search for candidate genes relevant to human ASD, using whole genome sequences from patients with ASD. Transgenic mice with ablation of target genes, such as *Mecp2, UBE3A, NLGN3/4, CNTNAP2, SHANK3*, and *CTNND2* (38), display a substantial list of abnormalities in brain anatomy and physiology as well as behavioral modifications, providing valuable insight into neurophysiological mechanisms in human ASD (29, 39).

### Research Strategies Based on a Validation of Animal Models

An ideal animal model for any human psychiatric disease would typically meet the requirements for three standard criteria of model validation (40, 41). The face validity for ASD models is manifested in the behavior of the animal model that mimics the components of behaviors defined in human ASD. The construct validity addresses biological mechanisms underlying the symptoms of ASD. Finally, the predictive validity is based on pharmacological responsiveness as a disease model (42, 43). Since ASD is defined solely by behavioral modifications, mouse models of ASD are required to at least exhibit behavioral impairments that mimic ASD-phenotypes in humans.

Research strategy in genetic-driven models is composed solely of construct validity, in which mouse models exhibit a specific genetic mutation that is associated with human ASD. Other validations regarding mouse models of ASD including the underlying neural mechanisms that are also involved in the construct validity and the pharmacological responses representing predictive validity, remain to be investigated (44). A major challenge for genetically driven models of ASD is to obtain a compelling analog to human behavioral symptoms in ASD relevant to face validity, which would in turn shed light on identifying common neurobiological pathways or circuity closely relevant to the core symptoms of ASD. This process for the validation of an animal model of ASD is the standard for the majority of translational studies in which the elimination of candidate genes relevant to ASD is implemented in transgenic mice, and is verified by the assessment of behavioral modifications that mimic the core symptoms of ASD. Although there is some degree of agreement that these behavioral changes associated with ASD can be monitored in various mouse models by using specific test paradigms that were developed to measure such behavioral modifications, the fact that the behaviors of mice are substantially different than those in humans is something to consider, and attention must be paid to appropriately interpret mouse behavior.

### Behavioral Characteristics in Mouse Models of ASD

**Figure 1:**
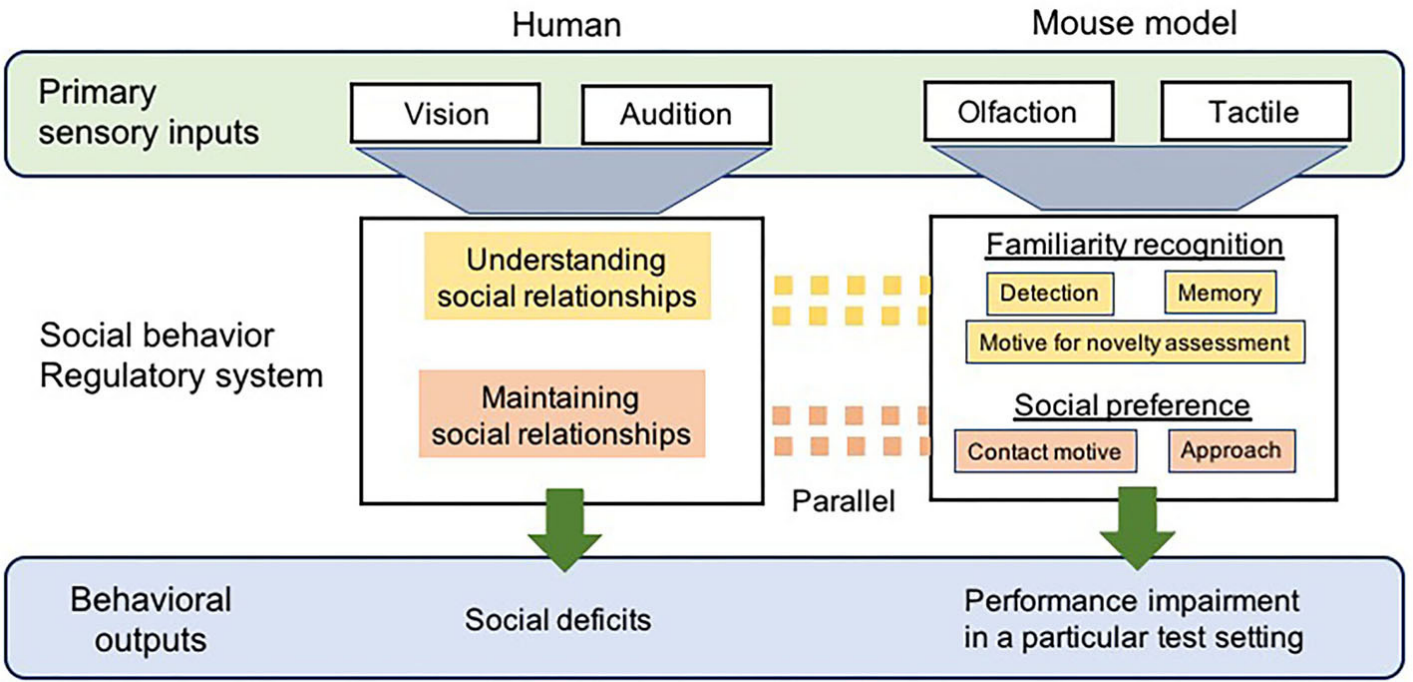
A diagrammatic representation of the comparison of social cognitive processing between human with ASD and ASD-mouse models. Sensory processes differ between humans and mouse models, primarily via vision and audition vs. olfaction and tactile, respectively. The regulatory neural circuitry/systems for social behavior that receives information from sensory systems, in both humans and mouse models, would be considered parallel to each other, in which recognition of familiar individuals and social preference required for the appropriate social interaction are regulated. As a result, the behaviors of social deficits representing ASD-like symptoms, including social interaction, language, and motor behavior, are also observed in humans diagnosed with ASD. Social impairments are measured by monitoring the behaviors of ASD-mouse models with using specific test paradigms.

It is difficult to identify the mouse models most suitable for ASD in humans, since the validity of transgenic mouse models is solely based on behavioral changes, and the behavior of mice is distinct from that of humans (45). Specific behavioral assays developed for mouse models of ASD have facilitated the discovery of fundamental principles that govern neural circuitry and mechanisms relevant to ASD-like behavioral symptoms in mouse models (40, 43). One mandate for successful translational animal models for a psychiatric disease is that they must account for species-specific/typical differences in the construct of interest. Therefore, we must understand what primary factors regulate social behaviors in mice and identify the factors expressed as social deficits for suitable ASD mouse models (Figure 1).

Mice are highly social animals, living substantial parts of their lives in groups, in which they use complex ways to communicate with each other to form social relationships, including social hierarchies, cooperative relationships among close relatives, and social bonds with partners (46, 47). Laboratory mice maintain most innate traits from their ancestors (48); thus, they express several behaviors that stem from adaptation to a natural environment (49). Such complex group-living requires multi-formal expressions of social interactions in appropriate ways, including an understanding of the social rules and friendships within certain groups (50), and expressions of proper behavioral responses in a situation-dependent manner. Therefore, social behavior comprises appropriate behavioral responses based on information about social features that requires the perception and integration of social cues via a complex cognition process that involves attention, memory, motivation and emotion (2, 51). Information from a range of senses can be used for discriminating familiar from unfamiliar conspecifics. This process must be dynamic and flexible, since social context continuously changes and is updated with new information.

Laboratory rodents (i.e., mice) are nocturnal and thus macrosmatic, and primarily use olfaction along with tactile senses for adaptive behaviors and survival (46, 51). Olfaction is a major modality through which rodents detect and identify potential social partners via volatile signals to determine whether the individual signal recipients display approach behavior to engage in further assessment (52). In the contact range, rodents exchange olfactory signals by directly sniffing the anogenital area to detect non-volatile exocrine compounds, in conjunction with tactile palpation via the whiskers to gather additional social information and to express acceptance of social contact with each other if possible (46, 53). Olfaction is crucial in mouse behaviors essential for successful group living, including identification of predators, distinguishing of familiar individuals from strangers, and identification of individuals within the social hierarchy (47, 52). Odor information is perceived via signal transmission from the main olfactory epithelium and the vomeronasal system to the olfactory bulb and several regions in the brain, including the amygdala, for further processing of signals (54, 55). In addition to olfactory processes, tactile palpation using whiskers, particularly during facial investigation, and body contact are the key mediators of social interactions. Tactile information via the whiskers follows the somatosensory pathway from the ipsilateral brainstem trigeminal nuclei to the contralateral thalamus through the somatosensory cortex (56, 57). Mice also use auditory signals for social communication, including emitting distress calls and a variety of ultrasound vocalizations, which require the activation of a corticostriatal neuronal circuit (58, 59).

Humans are not highly dependent on odorant cues or tactile inputs while making decisions regarding social interactions; thus, this undoubtedly appears to be a significant caveat for using mouse models. However, these multisensory modalities in rodents are essential to all aspects of social interaction including the recognition, assessment, and reception (acceptance) of a potential social partner, which directly activate neural pathways coupled with the expression of social behavior (2, 60) (Figure 1). The ability to distinguish between individuals via olfactory and tactile cues in order to identify familiar individuals is vital for their social behaviors, including formation and maintenance of relationships with them (61, 62). Comparative studies have pointed out that the neurobiological mechanisms underlying social behavior are highly conserved across species, regardless of differences in their sensory processing (63). Parallels exist in the neural pathways/circuitry supporting social communicative behaviors between humans and rodents (45) (Figure 1). A proper understanding of the functions of the sociosensory and cognitive processes in rodent models may lead to a reliable interpretation of behaviors that has substantial implications for human health. Preclinical rodent models for the study of social behavior pertaining to ASD must undergo a comprehensive analysis to assess the variables involved in social recognition (via initial olfactory detection), the assessment of potential social partners, and subsequent receptive behaviors (based on preference) during a bout of olfactory and tactile social engagement.

### Tactile Phenotypes in Human ASD

Atypical sensory experience is reported to occur in as many as 90% of ASD patients (64, 65) and has been shown to affect every sensory modality including vision (66), audition (67), taste (68), touch (69, 70), and smell (71, 72). These sensory symptoms have been highlighted in early reports but as secondary aspects of ASD rather than primary phenotypes (73, 74). Neurobiological alterations accompanied by sensory symptoms in ASD have been called to attention for understanding the mechanisms underlying ASD (75). Furthermore, clinical implications indicate that perceptual symptoms in ASD patients are evident early in development, and thus, exhibit a potential for shedding light on early diagnostic markers (74, 76). It is still unclear whether sensory issues in ASD result from long-term social deficits or are a result of domain-primary mechanisms that affect social and cognitive deficits.

Recent reports have indicated that approximately 60% of individuals with ASD exhibit altered tactile sensation (65, 69). Some individuals display self-injury by skin picking and self-biting (77, 78) and excessive responses to touch and pain (79–82). However, the polarity of tactile abnormalities in ASD is unclear, as some individuals simultaneously express hyper- and hypo-sensitivity to tactile stimuli, often depending on the context and stimuli (83–85). The role of pain sensitivity and the mechanisms underlying tactile abnormalities are also poorly understood.

The sense of touch is essential for sensorimotor exploration and control of the environment, and touch is a major component of social interactions especially during early development. Touch communication between mothers and infants facilitates physiological development (86) as well as cognitive and motor performances in infants (87, 88). Therefore, tactile abnormalities may contribute to avoidance of social touch via an inadequate amount of touch information produced by the peripheral nervous system or maladaptive circuit formation in the central nervous system (89, 90). The importance of affective touch through development and in social communication leads to the hypothesis that a problem in somatosensory processing may contribute to primary symptoms or core mechanisms of ASD.

The sense of touch, especially affective touch, is mainly processed via the stimulation of low-threshold mechanoreceptors (LTMs) expressed in the skin and joints (91) containing the end organs of the Meissner corpuscles (92). LTMs are innervated by myelinated Aβ afferent nerves that allow fast and rapid reaction to a touch stimulus (91). Hairy skin also contains fewer encapsulated LTMs innervated by a class of unmyelinated low-threshold mechanosensory nerves known as C-tactile afferents in humans (93). While the rapid first touch system is beneficial for detecting (and thus, protecting from) potentially harmful and threatening stimuli (e.g., pain, thermal, and itch- related) with advantages for survival, the slow second touch system is also useful for providing detection of gentle touch sensations via sensitive afferent C fibers, such as social touch (91). Although research on peripheral touch sense in ASD is still ongoing, the peripheral mechanisms are known to be orchestrated by the central pain/touch system, including endogenous opioid processes (94, 95) and the hypothalamic hormonal system (cf. oxytocin) (96, 97), and plays a potentially key role in ASD (98, 99).

Deficits in peripheral somatosensory processing does not necessarily cause impairments in certain higher-order cognitive functions, such as repetitive behaviors and memory deficits, that are commonly observed in ASD. Genetic mutations associated with ASD symptoms may contribute to both peripheral and central nerve issues leading to complex effects on the spectrum of behavioral deficiencies (57, 100). Sensory symptoms in ASD, including tactile abnormalities are not restricted to somatosensation or touch-pain responsibilities (79). Abnormalities in tactile sensations could lead to abnormal social behaviors. For example, tactile abnormalities may contribute to cognitive symptoms, such as anxiety, attention deficits, and sleep deficits, since somatosensation is closely associated with uninterrupted searching and detecting external stimuli that are possibly relevant to threat or danger (101). In mouse models, tactile impairments induced by transgenic mutations result in not only deficits in tactile-relevant behavioral performances, but also in impaired social approaches in a social interaction setting (56, 102). ASD research using transgenic mouse models requires two different directional investigations: (1) how sensory abnormalities, including deficits in somatosensation, affect the performance of social behaviors that are relevant to the core symptoms of ASD, and (2) how social behaviors of transgenic mouse models with ASD mimic the core symptoms of ASD in humans. We will review the recent literature on tactile abnormalities in ASD mouse models, focusing on the association between tactile phenotypes and social impairments. Thus, we will illustrate a potential pathway of how tactile abnormality is associated with deficits in social behavior.

### Impairments in Tactile Senses in Mouse Models of ASD

The sense of touch in mice is essential for somatosensorimotor control and for recognition of their environment, thus promoting survival, and plays an integral role in forming intimate relationships that are key for sustaining neurodevelopment and social behavior (103). Social touch, particularly during development, is critical for maintaining mother-infant relationships (104), thus, regulating a mother's contact and care with pups as well as the physiological and behavioral development of infants (105, 106). The significance of affective touch during development implies that abnormalities in somatosensory processing in ASD mouse models may be involved in the malformation of neural structures during early life, which may also be responsible for social deficits (91).

There are several mutant mice with dilution of specific target genes associated with human ASD. Although these mouse models are mainly evaluated by abnormalities in social behavior, an increasing number of reports illustrates tactile performance and impairment in these mice (103, 107).

#### Fmr1

The fragile X mental retardation 1 locus (*Fmr1*) resides in the X chromosome, and codes for the fragile X mental retardation protein, which has been implicated in synaptic protein synthesis and synaptic plasticity (108). *Fmr1* knockout (KO) mice have been shown to present structural abnormalities including abnormalities in dendritic morphology and protein synthesis, although different traits depend on the background strain (109), and display hypersensitivity to whisker-related tactile stimuli (108, 110), which has been linked to the abnormalities in the somatosensory cortex (110). A blunted tactile response in *Fmr1* KO mice is associated with touch insensitivity during the critical period (first 2 weeks of life) of development in which the neural connections from whiskers (tactile) to thalamocortical pathways are formed (111).

#### Mecp2

Rett syndrome, an X-linked disease that affects girls, is caused by mutations in the gene encoding for the methyl-CpG binding protein 2 (*Mecp2*), which remarkably influences gene expression in neurons (112). Because of the significance of *Mecp2* genes in neuronal function, *Mecp2*+/– females are used as a model for Rett syndrome (113, 114), and exhibit a variety of neuronal malformations, including reduced size of the cell body, cortical layers, and spine densities (112, 115). Rett syndrome involves somatosensory abnormalities, including hyposensitivity to tactile stimuli and blunted pain sensitivity (116, 117). Despite the involvement of *Mecp2* in somatosensory processes, a rat model with *Mecp2* dilution demonstrated an unclear direction of pain/tactile modification depending on the type of tactile stimuli (118). Mice with early postnatal dilution of Mecp2 genes consistently exhibited impairment in whisker-related tactile abilities (57, 100).

#### TSC1/TSC2

Mutations in either the *TSC1* or *TSC2* gene, which are both associated with the construction of critical astrocyte structures cause tuberous sclerosis complex (119). Because homozygous mutants cannot survive the embryonic period, heterozygous mutants are used (119) and mice with *TSC1* deletion at the prenatal stage have been shown to display exaggerated grooming that is associated with abnormal tactile sensation along with malformation of thalamocortical circuitry (120). While these genes are widely expressed in central and peripheral tissues, mice lacking *TSC2* genes specifically in peripheral sensory neurons exhibited normal tactile sensation to cold and mechanical noxious stimuli, but enhanced pain sensitivity to heat stimuli (121).

#### SHANK3

Deletion of the human *SHANK3* gene near the terminus of chromosome 22q in mice results in turn down of all isoforms with multiple promotors (122, 123); thus, several mice with the deletion of different isoforms of *SHANK3* have been generated. Studies across a variety of *SHANK3* heterogeneous mice have documented deficits in glutamatergic transmission (122, 124) and reduced pain sensitivity (123, 125). *SHANK3* is broadly expressed in the dorsal root ganglion (DRG) neurons and spinal cord both of which regulate pain transduction. Thus, mutant mice with the deletion of *SHANK3* exhibit impaired heat hyperalgesia (125). Whisker-related tactile hy*per-sen*sitivity is observed in *SHANK3* mutant mice due to dysfunction of cortical GABAergic interneurons in the primary somatosensory cortex (126).

#### CNTNAP2

A recessive non-sense mutation in the Contactin-associated protein-like 2 (*CNTNAP2*) gene causes a syndromic form of ASD (127). The *CNTNAP2* variant leads to abnormal functional connectivity in humans and reduces the number of interneurons in mice with dilution of the *CNTNAP2* gene (128). Mice lacking *Cntnap2* genes have demonstrated enhanced pain reactivity to several noxious stimuli (129).

#### UBE3A

Angelman syndrome is a severe neurodevelopmental disorder caused by a mutation in the maternal *UBE3A* allele (130, 131). The *UBE3A* gene is primarily expressed in the central nervous system (132) and commonly induces sensorimotor impairments in patients, including malsensitivity to pain stimuli (133, 134). Mice with dilution of *UBE3A* maternally exhibit enhanced pain responses accompanied by abnormalities in the DRG neuronal formation (135). However, the enhanced pain response in mice lacking the *UBE3A* gene may be responsible for sex differences, since male mutant mice exhibited an enhanced pain reaction while female mutant mice rather displayed a heightened tolerance to thermal pain stimuli (136).

Given the inconsistency of the genetic effects relevant to ASD on tactile and pain sensitivities, more work is needed to determine whether the loss (or modification) of control in tactile and pain sensations stems from the dysregulation in the peripheral nervous system or in neural projections to somatosensory circuits from the brain stem to the thalamocortical network. Furthermore, it is crucial to pursue to elucidate the mechanisms by which impaired tactile senses affect developmental milestones of behaviors and, eventually, the performance of social behaviors relevant to the core symptoms of ASD.

### From Somatosensation to Social Behavior

In rodent models, tactile information received through exquisitely sensitive whiskers plays a critical role in survival via exploring and assessing the external world (137, 138). Throughout development, tactile perception through whiskers serves as a significant tool of communication with the dam and siblings (139, 140). Whisker trimming during the neonatal stage leads to deprivation of primary tactile senses, resulting in malformation of thalamocortical somatosensory circuits (141, 142). Neonatal whisker trimming (e.g., daily for 2 weeks from postnatal days 1–3) induces delayed/modified motor development (140, 143) and causes long-lasting behavioral modifications (138, 144). Rats with whiskers trimmed during the neonatal stage exhibit enhanced exploratory behavior in a novel environment (145, 146), possibly due to impairment of tactile perception. Mice with whiskers trimmed during the neonatal period also showed decreased social approach in the 5 min sociability test (147), indicating a close linkage between a deficit in tactile experience and expression of social behavior. However, in the same sociability test, an impairment in social approach was also found when the whiskers of adult male mice were temporarily trimmed (147). This can be attributed to a temporary defect in the whisker-mediated tactile sense, resulting in exaggerated exploratory behaviors in a novel environment and impaired detection of social stimuli in the sociability test. A temporary loss of whisker sensation indicates that the tactile exploratory component via whisker palpation is significantly involved in the regulation of social approach behavior.

Whisker palpation, particularly during social interaction, is a key mediator of social behavior, followed by olfactory processes (148). During close-range social interactions between mice, it is observed that facial investigation of other mice induces whisker palpation and muzzle sniffing, whereas anogenital investigation by sniffing non-volatile components of odorants typically induces a flight response (~30%) in the other mouse (102, 149). Mice with malfunctioning whiskers, caused by temporary whisker-trimming, clearly displayed a reduction in facial investigation during direct social interactions (56, 102). This is consistent with the sparse social investigation performed by mice with temporary whisker-trimming (150). Somatosensation, apart from whisker sensation, is also involved in a particular social response during social interaction. Mice with genetically nulled body sensations (adenylyl cyclase type 1 knockout mice) displayed a robust flight response (>70%) to social contacts with their counterparts, whereas wild-type mice with clipped whiskers did not show such a response (102). These tactile phenotypes illustrate the significance of whiskers, particularly in facial investigation during the initial phase of social interactions, and the importance of somatosensation as a significant component of the subsequent social interactions, such as contact reception.

Huddling with familiar conspecifics is common among most mammals to maintain prosocial (e.g., friendly) relationships (104, 151), and is regulated mainly by thermal (thus, tactile) contacts (104). Particularly in neonates, huddling reduces the metabolic costs of physiological thermoregulation (104, 152). However, rodents continue to huddle into adulthood, forming an olfactory preference for a warm soft touch (151). Although this is a theoretical leap from rodent huddles to the distinct attachment behavior in humans, such as “cuddle” contacts, empirical evidences in rodent models illustrate that this strong preference for huddling is linked to the motive of bodily contact with familiar conspecifics, which may be supported by neural systems, such as hypothalamic oxytocinergic circuitry, common across species (152, 153). Therefore, prosocial behavior between mice involves achieving physical contact with their familiar partners and acceptance of contact with each other. These motives for familiarity-dependent social interest and preference demonstrated in mouse models can be considered representative of the core behavioral symptoms of ASD in humans.

### Factors Underlying Social Deficits in Mouse Models of ASD

Social behavior in mice entails a variety of patterns that can be segregated into both negative (e.g., aggression, avoidance, and social anxiety) and positive (e.g., prosocial contacts, social bonds, playful interaction) interactions (48, 154). Appropriate ASD models should involve behavioral phenotypes associated with the core symptoms of ASD, such as a lack of social interest and inappropriate social interactions as well as restricted and repetitive behaviors (36, 44). Studies using mouse models of ASD have usually focused on one specific domain of positive social behavior that is rigorously controlled and extracted from more naturalistic and complex social behaviors that include all domains of behaviors between animals (154). There are several types of specific measurements for examining the underlying mechanisms of social behavior in mice that have been developed to mimic ASD-like behavioral phenotypes, including social recognition and social preference.

Most of these behavioral paradigms exploit the innate drive in mice to exhibit spontaneous investigatory behavior toward social stimuli (e.g., conspecifics), and are used to monitor distance-based behaviors toward social stimuli. The major question raised in this chapter is whether the behavioral measurements of mouse models that stem from the investigatory drive toward social stimuli can represent the core behavioral symptoms with ASD without any theoretical flaws or technical errors in their interpretation. As outlined below, we will discuss the validity of social behavioral tests that are designed to measure certain aspects of social deficits associated with ASD, with a focus on the parametric factors underlying behavioral measurements obtained from each test paradigm.

### Primary Factors Mediating Social Recognition

A social recognition test is used to assess the ability of test animals to discriminate between social stimuli based on social memory (e.g., familiarity), which allows the identification of each conspecific in social living. Social recognition of rodents heavily relies on the detection and discrimination of olfactory cues emanated from conspecifics (155, 156). The sources for the odor (chemosensory) signals used for social recognition include body fluids such as urine, feces, and secretions from the skins or specific scent glands relevant to pheromonal signals (52, 62). There is compelling evidence indicating that individual unique compositions of these odors act as an olfactory signature (46), achieving individual recognition by discriminating volatile and non-volatile components of chemosignals. As a result of odor detection, mice (as well as rats) exhibit scent marking behaviors where they deposit scent marks and detect them in the environment. These scent marks underlie natural communicative interactions regarding social status, such as territoriality, dominance, reproductive status, and health and nutritional conditions (47, 52).

With regard to the anatomy of the procedure for a social recognition test, the test relies on the exposure of subject mice toward conspecifics as a stimulus animal and on monitoring the duration that subject mice spend in investigation or in proximity to the conspecifics, which is referred to as a social memory measurement. It is hypothesized that mice possess a motive to investigate/sniff ‘unfamiliar' social cues, such as odors, that they encounter; thus, the subject mice would spend more time investigating social cues if they are unknown or unfamiliar, while they would spend less time if they are familiar.

Several modifications to social recognition tests have been made, including the habituation/ dishabituation task (157) and the social discrimination paradigm (53, 158). The habituation/dishabituation task is one of the most widely used methods for investigating social recognition. In this test, subject mice are exposed over several trials to a social stimulus that is initially unfamiliar. The duration of each trial is typically 1–5 min, and the mice are allowed to investigate by coming into contact with, or sniffing, a social stimulus as part of an innate novelty investigation. These exposures to the same social stimulus are separated by intervals that could be set up for a short period (e.g., 1 to 10 min) or a long-term period (e.g., 1 to 24 h) based on the memory performance that the investigator attempts to elucidate (62). The investigation time declines upon over trials, since the familiarity toward the stimulus animal increases with habituation. Following habituation, when investigation time has reached a plateau, the stimulus is changed to a novel (e.g., unfamiliar) social stimulus. The presentation of an unfamiliar stimulus at this point is expected to reinstate the investigation to initial levels if the subject mice can detect a difference between the previous and current social stimuli, indicating dishabituation.

Despite the usefulness of this test for assessing familiarity-based social memory performance, there are several difficulties in data interpretation due to repeated testing of the same subject mice possibly leading to non-specific, general habituation to testing procedures (158). The social discrimination paradigm, another version of the social recognition test, was developed to compensate for the weakness of the habituation/dishabituation task, consisting of just two sessions to assess social memory performance (53). However, a more important question to be addressed with regard to a test procedure for ASD-related behavioral phenotypes is whether the behavior of mice that is exhibited during the test would reflect certain characteristics of the core symptoms in human ASD. The performance of subject mice in this test paradigm primarily relies on the investigatory drive to social novelty (unfamiliarity), by which the mice must express a heightened amount of investigatory sniffing in response to a social stimulus during a short period of time (1–5 min). Therefore, the mice should maintain intact olfactory ability in the detection of unfamiliarity and an intact motive to approach and investigate unfamiliar social stimuli.

The identification of unfamiliarity via olfactory cues is processed by two different olfactory pathways, via the detection of volatile and non-volatile odor chemicals (55, 62). It is likely that mice can exhibit distinct social responses to conspecifics by having access to the volatile components of odor, while rats appear to require access to non-volatile components to exhibit odor recognition (54). The screening processes via olfactory cues also depend on the type of odor components that the odor recipients diagnose upon detection. For example, rodents (both mice and rats) have an ability to discriminate healthy from sick animals, to avoid potential parasites or disease contagion (159), via the detection of volatile components (52). It is poorly understood whether transgenic mice with target gene mutations associated with ASD have a deficit in olfactory processing of volatile or non-volatile components; more attention should be paid to this concern [e.g., (52, 54)].

In the social recognition paradigm, exploratory (sniffing) behavior exhibited during the 1–5 min sessions of social encounters would be mainly mediated by the motive to assess uncertain stimuli and environment, as a form of novelty assessment (160, 161). From an ethological perspective, the investigatory drive for novelty assessment is essential for searching and elucidating potential threats that animals may encounter (160). Therefore, the investigatory behavior of a mouse when it is confronted with a social stimulus is interrupted by a heightened anxiety in response to novelty or aggressiveness to conspecifics (162). Direct physical contact with stimulus animals, if present, may induce aggressive feedback that accelerates anxiety responses and thus decreases investigatory drive. To avoid this, a young female or juvenile animal is usually used as a stimulus animal; this factor is highly relevant to experimental reliability (163). A lack of social motive to approach and contact with (cf. particularly familiar) conspecifics may be linked to a core symptom of human's ASD if these mice display intact olfactory ability. However, a decreased novelty investigation (e.g., risk assessment) caused not by a lack of social motivation, but heightened anxiety or a lack of defensive behavior would also occur in the social recognition test, which may be mediated by different putative drive and neural mechanism from those associated with ASD symptoms. A heightened sense of anxiety and inappropriate social interactions are also involved in the behavioral phenotypes of human ASD; thus, it is difficult to assess whether an abnormal expression of exploratory behaviors in mouse models of ASD during a session of the social recognition test may interfere with underlying behavioral symptoms associated with ASD.

### Primary Factors Mediating Social Preference

There is a growing need for suitable behavioral tasks that measure social recognition and social motives or preferences in mouse models that are independent of novelty investigations, for translational studies on behavioral symptoms of ASD (40). The sociability test, also called the three-chamber test, was developed by Crawley (42) and has been widely employed to evaluate several fundamental aspects of mouse social behavior relevant to ASD symptoms, including social approach and preference, and social novelty discrimination (40). The three-chamber apparatus consists of two equal-size chambers that are separated in half or connected to a small center chamber. Two inverted holding cups or wire- grid bins are placed on the wall of each chamber compartment. In the test phases, the subject mice are offered a choice between chambers containing a holding cup; first, empty vs. a stimulus mouse, and second, a previously exposed mouse vs. an unfamiliar mouse (51).

**Figure 2:**
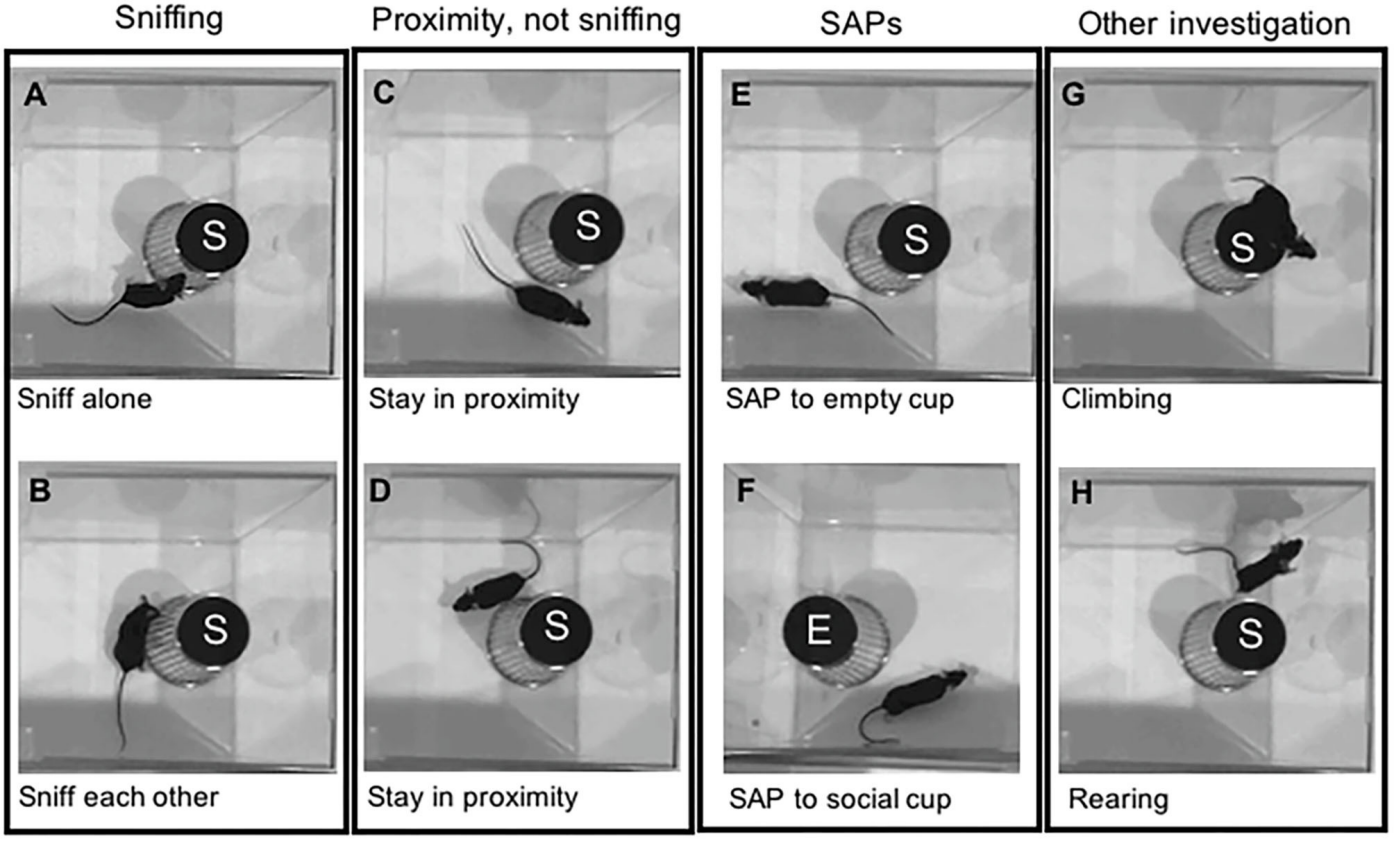
Graphical representation of mouse behaviors during the social preference test session. The test mice were exposed to an empty cup (E) and a cup containing a stimulus mouse (S). During the social preference test, test mice express a variety of investigatory behaviors toward a stimulus mouse, including **(A)** Sniffing the social cup; **(B)** Sniffing each other along with a stimulus mouse; **(C,D)** Staying in proximity of the cup; **(E)** Stretch attend postures (SAP) near an empty cup, and **(F)** SAP near the cup containing a stimulus mouse; **(G)** Climbing on the cup containing a stimulus mouse; and **(H)** Rearing near the cup containing a stimulus mouse. All these behaviors, except **(F)**, would be considered measures of social preference time in the standard sociability index.

During the sessions, the subject mice are allowed to move freely through the test chambers, and the sociability score refers to the ratio of investigation (visiting) time of these cups/chambers. Similar to the social recognition test, the investigation involves time spent in proximity of the cups (usually 3–5 cm distance) or visiting the chamber in which the stimulus mouse was contained, which implies (1) no requirement of direct contact with the stimulus animals or even the holding cup containing the stimulus animal and (2) no discrimination of behavior types that the subject mice exhibit in the proximity of the mouse cup. These simplified methods to calculate sociability scores can achieve a rapid, automated data collection to facilitate research productivity, but at the same time, with sacrificing data reliability with the appropriate interpretation of behaviors of the subject mice. Blocking direct contact or interactions with the stimulus animal provides discrete control of the behavior of the test subjects, but restricts the behaviors, thereby allowing them to express only olfactory-based investigations (Figure 2).

The holding cup that contains the stimulus animal restricts direct access of the test animals to the stimulus animal, preventing any observable “social interaction” in this situation. The stimulus animal is still capable of moving in the holder but is restricted from reacting with appropriate postures and forms. Considering the importance of interactive processes through sensory modalities, including non-volatile odorants and whisker and body tactile exploration (46, 48), this “window shopping”-like procedure restricts mice to assess a stimulus mouse only via volatile odorants and vocalization. Accordingly, odorant signals convey a variety of individual specific information that is able to strongly modify the behaviors of odor recipients, such as pheromones (46, 164). Familiarity-related olfactory cues strongly modulate the assessment behavior of odor-recipients (61, 165), and the discrimination of these familiarity cues is required for the appropriate expression of social preference. However, it is disputed whether such investigation/assessment behavior during the test can represent the core symptoms of ASD, or simply initial novelty assessment.

Interestingly, an inbred strain of BTBR+ltpr3^tf^/J (BTBR) mice characterized by autism-like low sociability (166, 167) produces volatile olfactory cues that induce avoidance (social withdrawal) in other mice, including those belonging to the same BTBR strain (148). Detailed analysis of the behavior of a BTBR subject mouse toward a BTBR stimulus mouse indicated that BTBR subject mice exhibited a certain amount of sniffing toward the stimulus mouse during the initial few minutes of the sociability test, consistent with the intact drive for novelty assessment. Thereafter, they strongly avoided the BTBR stimulus mouse, but not stimulus mice of other strains (e.g., C57BL/6J and BALB/cJ) as an aversive response (148). Such avoidance (time empty > social) in the sociability test has been reported in other genetic-driven ASD mouse models, such as Shank3 (168) and 4E-BP2 mutant mice (169). These findings imply that discrete social approaches in subject mice, at least in the initial few minutes, represent a novelty assessment that is not only determined by memory (i.e., the familiarity of stimulus animals) but also by the olfactory cues from the stimulus animals and perceived by the subject mice.

### Determinants of Prosocial Behavior in Mouse Models

The nature of social behavior is based on the sequences of interactive behaviors between animals. First, the detection of (e.g., volatile) odorants activates the assessment of social features, which are determined by a motive for novelty assessment. Further investigation is then expressed as close assessment behaviors, such as approach and sniffing (e.g., contact) toward potential social stimuli, resulting in responses from the social stimuli if applicable, such as approach-withdrawal and sniff-contact behaviors (47). During this close interaction, exchanging social information through facial and anogenital (sniffing and whisking) investigation leads to further assessment of social features. These initial assessment phases are stabilized mainly via olfactory and tactile interactions with each other, leading to the determination of subsequent strategies for social interaction within a few minutes. The investigatory sniffing of an unfamiliar stimulus mouse by the subject mice decreases quickly within 5 min of observation, as corroborated by the social recognition and the sociability tests (148, 149, 170).

**Figure 3:**
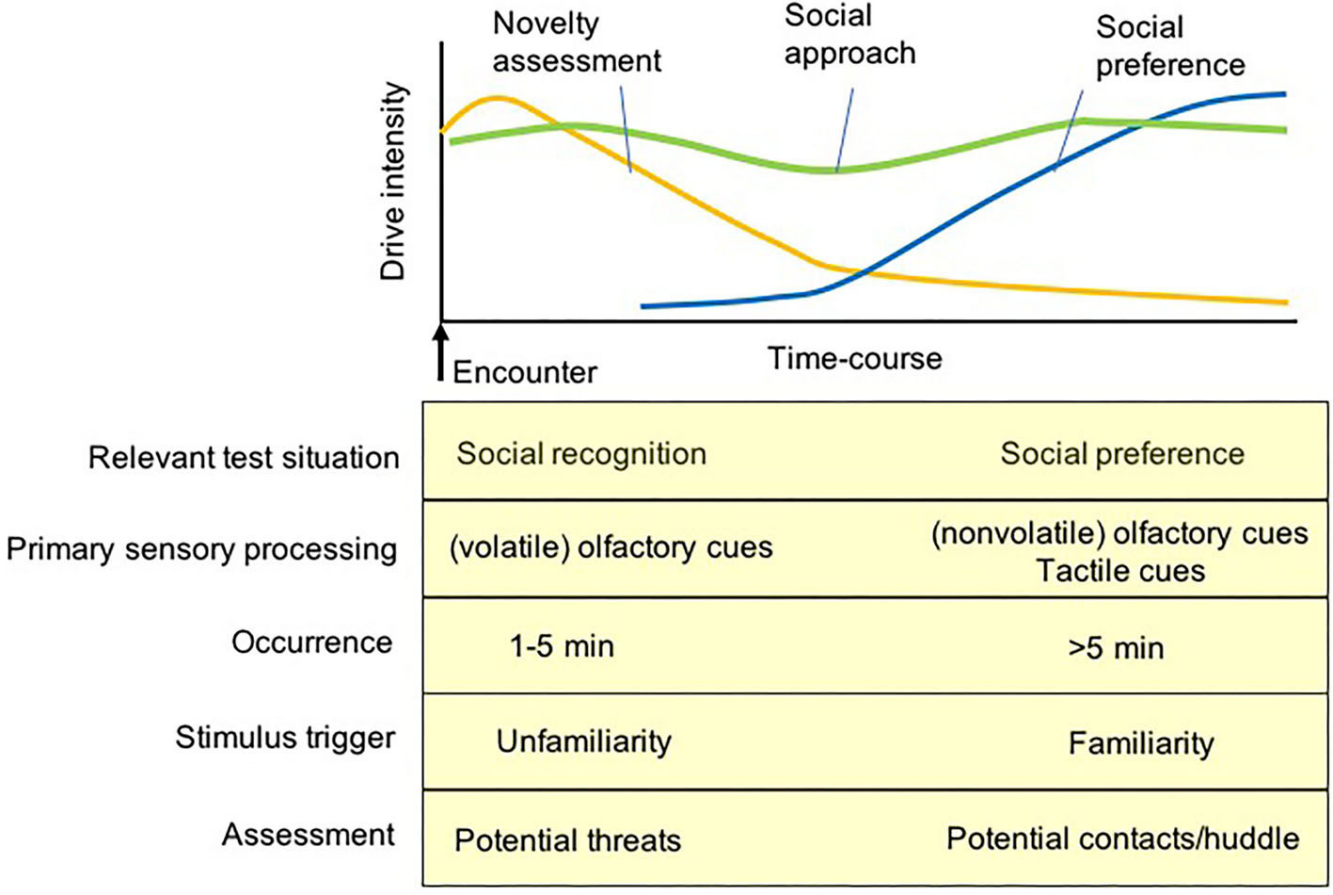
A diagram for putative internal drives of test mice primarily activating social approach/investigatory behavior during the social preference test. In the initial few minutes of the social recognition and social preference test, the approach behavior of test mice was primarily regulated by the drive of novelty assessment mediated by the olfaction of volatile cues to assess potential threats of an unfamiliar stimulus and environment. After the novelty assessment, the other social approach behavior of test mice is activated by the drive of social preference (as a compensation) regulated by contact-based behaviors via the processing of tow sensory inputs, non-volatile olfactory cues and tactile cues, to achieve physical contacts/huddle with a (familiarized) stimulus mouse.

Following the initial assessment phase, mice become familiar with each other and tend to remain in close proximity if physical contact is acceptable and permitted (41, 165). A long-term observation of social behaviors between initially unfamiliar mice in a semi-natural environment demonstrated that active social interactions such as sniffing, chasing, fleeing, and following are displayed initially, and are gradually displaced by actions such as huddling, where the animals stay in close physical contact (171). A predominance of huddling along with silencing of other active interactions represents prosocial relationships between familiar mice, which accounts for their drive for physical contacts with partners (165). Thus, the behavior displayed by subject mice in sociability tests can be illustrated as a time-course dependent process. The drive for novelty assessment is a primary determinant of the behavioral measures that stabilize ‘unfamiliarity' recognition mainly via olfactory cues of a stimulus mouse during the initial phase of testing, and the subsequent determinant of the behavioral measures is the drive for social contact expressed via huddling with conspecifics if available. This transition of strategies from novelty assessment to social contact is postulated to have occurred for the processing of social investigation displayed in these social behavior tasks (Figure 3). The initial phase of novelty assessment requires the assessment of the safety and familiarity of social stimuli, while the second phase of social contact requires establishing prosocial relationships with each other, such as huddling and similar physical contact. When direct physical contact is blocked by a (wire-grid) holding cup containing a stimulus mouse, the subject mice often exhibit investigatory behaviors to the mouse holding cup (e.g., biting, climbing, and sniffing) in order to achieve physical contact with the stimulus mouse or even to attempt to remove the intervening holding cups (51).

A careful observation of the types of behaviors expressed by the subject mouse when they investigate the holding cup containing a stimulus mouse, may provide some complements to the interpretation of behaviors of the subject mouse during the test. If freezing (e.g., immobile sustained back with a straightened tail) is exhibited in the proximity of the holding cup (51, 160), the behavior should be interpreted as a typical defensive reaction to a potential threat in the test chamber; thus, distinguishable from those driven by social contacts. If assessment behaviors such as stretch-attend postures (160) or sniffing (or biting) the holding cups (even climbing on the cups) are evident (Figure 2), they would be interpreted to be driven by novelty assessment or an attempt to release a stimulus mouse from the holder (172, 173), which could be distinguished from the social contact drive. When the subject mice remain on the side far from the holding cup containing a stimulus mouse, lower social interests would be applicable, but in some cases, a heightened aversion to a stimulus mouse as an avoidance response may also be interpreted to have mediated this behavior (Figure 3).

The measurements of the sociability test rely simply on the distance-based assessment of a social stimulus, omitting the interpretation of behaviors displayed by the subject mice. We must take into account the time differences in the motivation for investigatory behavior (e.g., the novelty assessment and social contact) and other types of behavior exhibited by subject mice exposed to a social stimulus. Further investigation based on the analyses of behaviors with multimodal functions expressed by these mice will provide a better understanding of the behavioral characteristics exhibited by transgenic mouse models of ASD in the test chamber. For example, general and social olfactory abilities can be measured by separate behavioral tests, such as the olfactometer, odor-based habituation-dishabituation test (61), and odor discrimination test (174). Tactile sensation and somatosensation can be evaluated via other somatosensory assessments, such as the gap-crossing test, texture discrimination test, and Von Frey touch test (102, 148). Although these assessments of sensory processing in ASD mouse models would provide further understanding of their sensorimotor behaviors (45), we must separately take into account the features of sensory modalities of the core regulatory mechanisms in socially deficits animals underlying the behavioral performance of mouse models.

## Conclusion

ASD is a heterogeneous disorder, defined solely by behavioral deficits. Therefore, most animal models are based on loss-of-function mutations of genes associated with ASD and must be validated by the similarity of behavioral phenotypes with human ASD symptoms. Of particular importance is that we still need to develop a better way to measure and interpret behaviors of mice in the specific test settings for measuring ASD-like behaviors. Transgenic mouse models are popular for use in research strategies from genetic to behavioral, and they mostly maintain their innate trait for adaptation, including a nocturnal life style with macrosmatic modality processing. It is believed that the social behavior of mice relies primarily on olfactory cues to discriminate between familiar and unfamiliar conspecifics. Most behavioral tests developed for assessing ASD-like phenotypes in transgenic mice are based on this belief, particularly in olfactory-based investigations, and thus, the assessment of behavior mainly relies on the distance between the subject mouse and the stimulus mouse. In this course of experiments, the investigatory behavior of the subject mice is mediated by different processes in a stimulus (familiarity) and time-dependent manner. In the initial phase, the drive for novelty assessment is activated to elucidate the safety and familiarity of the stimuli and environment, and thereafter, another drive for social contacts dominates to facilitate approach and contact behaviors. These behaviors are regulated by multiple modalities including olfaction and tactile processes, although the contribution of tactile information in mice during the second phase of social behavior testing has been overlooked. Analyses of behavioral patterns and forms regarding distance to social stimuli and the sequence of behavioral expressions promise further understanding of the neuronal mechanisms underlying social deficits representing ASD symptoms. This knowledge provides the basis for the analysis of the sequential processing of multisensory inputs to generate social investigatory and approach (contact) behaviors, which will likely lead to an appropriate interpretation of behaviors that represent the core symptoms of ASD. With the proper strategy for behavior analysis and interpretation in transgenic mouse models, we can gain further insight into the brain areas and circuits modulating social behavior and thus social deficits associated with ASD.

## Author Contributions

HA is fully responsible for the content of the article and contributed to the submitted version.

## Conflict of Interest

The author declares that the research was conducted in the absence of any commercial or financial relationships that could be construed as a potential conflict of interest.
